# Head and Neck Lymphoma in an Iranian Population

**Published:** 2017-09

**Authors:** Nafiseh Shamloo, Alireza Ghannadan, Mahsa Jafari, Samane Ahmadi, Hamed Mortazavi, Maryam Baharvand

**Affiliations:** 1 *Department of Oral and Maxillofacial Pathology, School of Dentistry, Shahid Beheshti University of Medical Sciences, Tehran, Iran.*; 2 *Department of Pathology, Cancer Institute, Imam Khomeini Hospital Complex, Tehran University of Medical Sciences, Tehran, Iran.*; 3 *Dental Student, Shahid Beheshti University of Medical Sciences, Tehran, Iran.*; 4 *Department of Oral and Maxillofacial Medicine, School of Dentistry, Shahid Beheshti University of Medical Sciences, Tehran, Iran.*

## Abstract

**Introduction:**

This study is aimed to assess the prevalence and characteristics of head and neck lymphoma in a defined group of an Iranian population.

**Materials and Methods:**

In this retrospective study, 126,450 biopsy reports from two referral Pathology Departments, (Tehran, the capital of Iran) were evaluated. In cases with head and neck lymphoma, other variables such as age, sex, specific location of lesions, and histopathological findings were recorded. Descriptive statistics were used to measure the prevalence and characteristics of head and neck lymphoma by means of SPSS soft ware, version 18.

**Results:**

In total, 513 (0.4%) cases had head and neck lymphoma (46.9% male, 27.1% female) with a mean age of 46±6.2. Of the total lesions, 200 (0.15%) were Hodgkin lymphoma and 313 (0.25%) were non-Hodgkin lymphoma. Nodular sclerosis was the most common (62.5%) histopathological subtype among Hodgkin lymphoma. In non-Hodgkin lymphoma, diffuse large B-cell lymphoma (62.3%) had the highest frequency. In Hodgkin disease, classic Hodgkin lymphocytic rich, mixed cellularity, and lymphocyte depletion were only seen in the neck compartment. Bone involvement was only found in Hodgkin nodular lymphocytic predominant variation. In non-Hodgkin lymphoma, the tongue, palate, and vestibular mucosa were affected only by diffuse large B-cell lymphoma. Jaw bones were only involved with diffuse large B-cell lymphoma compared to other bony structures. T-cell lymphoma and mucosal associated lymphoid tissue lymphoma were also found.

**Conclusion:**

Diffuse large B-cell lymphoma is the most common subtype of non-Hodgkin lymphoma especially in the tongue, palate, vestibular mucosa, and jaw bones.

## Introduction

Head and neck cancers are the sixth most common worldwide malignancy with more than 500,000 new cases annually (1). The varying incidence range of 5% to 50% in different countries may be related to variations in socio-cultural characteristics, risk factors, study design, and data collection (1, 2). Lymphoma is the malignant neoplasm of lymphatic cells which constitutes the third most common malignancy in the head and neck region after squamous cell carcinoma and salivary gland tumors (3). In general, it is the tenth most common cancer in women and the eighth most frequent cancer in men. The incidence has been estimated to be 4.2 in males and 2.8 in females per 100,000 in developing countries, while in developed countries the incidence is 10.3 in males and 7 in females (4). 

Hodgkin and non-Hodgkin lymphoma (NHL) are the two main types of malignant lymphomas. More than 40% of NHL are extra nodal and comprise 5% of head and neck malignancies (3,5). 

According to literature, the head and neck region is one of the most common sites for extra nodal lymphomas with 1.5% to 8.8% of lymphomas arising in the oral and para oral regions (6-8). 

Although having a wide age range, NHL generally occurs during adulthood and rarely in childhood (with a peak incidence between 5-9 years) (3,9). In Hodgkin lymphomas, there are two peak incidences between 15 and 35 years and after 50 years (3). 

Head and neck lymphomas usually appear as an asymptomatic mass; however, they may be associated with pain, fever, and weight loss (3,10,11). 

There is a male predilection with a male: female ratio of 1.5:1 (12); however, no significant gender difference was noted by Urquhart (13). 

Albeit lymphomas are a roughly common entity in the head and neck region limited studies have been performed on an Iranian population in this regard. 

Therefore, the current investigation was designed as the first large series to provide a preliminary report on patients with head and neck lymphomas in Tehran (one of the largest three cities in the Middle East along with Istanbul and Cairo), Iran (14).

## Materials and Methods

In this multi-center, retrospective study, 126,450 biopsy reports over a 13-year-period (2002-2015) were retrieved from archives in the Oral and Maxillofacial Pathology, Shahid Beheshti Dental School and Cancer Institute of Imam Khomeini Hospital, Tehran, Iran. The inclusion criteria was a confirmed diagnosis of head and neck lymphoma as well as data regarding features of disease such as age, sex, specific location of lesions, and histopathological findings. Descriptive statistics were used to measure the prevalence and characteristics of head and neck lymphomas in terms of sex, type of lesions, and location by means of SPSS software, version 18.

## Results

In this study, out of a total 126,450 cancer biopsy reports, 513 (0.4%) cases had an established diagnosis of head and neck lymphoma with 241 (46.9%) cases being male and 139 (27.1%) female. Patients' age ranged from 6 to 90 years with a mean age of 46±6.2. Of the total lesions, 200 (0.15%) were Hodgkin lymphoma (126 (63%) male, 74 (37%) female) and 313 (0.25%) were non-Hodgkin lymphoma (193 (62%) male, 120 (38%) female). The mean ages of males and females diagnosed as Hodgkin lymphoma were 34.4 and 27.8 years, respectively. In non- Hodgkin lymphomas, the mean ages for men and women were 61.5 and 46.6 years old. 

Nodular sclerosis (62.5%) and lymphocyte depletion types (1%) were the most and the least common histopathological subtypes among Hodgkin lymphomas, respectively. In non-Hodgkin lymphomas, diffuse large B-cell lymphoma (62.3%) had the highest frequency and mantle cell lymphoma (0.6%) had the lowest frequency. The distribution of head and neck Hodgkin and non-Hodgkin lymphomas in terms of histopathological variations, sex, and involved location has been summarized separately in Table 1 and 2. In Hodgkin disease, classic Hodgkin lymphocytic rich, mixed cellularity, and lymphocyte depletion were only seen in the neck compartment and in almost all variations there was a male predilection. Bone involvement only was found in Hodgkin nodular lymphocytic predominant variation (Table.1). 

Non-Hodgkin disease in almost all sites of the head and neck was of diffuse large B-cell lymphoma subtype. Except for follicular lymphoma, males were more affected than females. However, there was no sex predilection in mantle cell lymphoma and mucosal associated lymphoid tissue lymphoma (Table.2). 

**Table1:** Distribution of gender, location, and histopathologic pattern among patients with Hodgkin lymphoma

**Pathology**	**Gender**		**Neck**	**Level 1 cervical ** **lymph nodes**	**Parotid gland**	**Bone**
Hodgkin nodular lymphocytic predominant	Male	16 (8%)	15 (7.5%)	1 (0.5 %)	-	-
	Female	8 (4%)	7 (3.5%)	-	-	1 (0.5 %)
	Total	24 (12%)	22 (11%)	1 (0.5 %)	-	1 (0.5 %)
Classic Hodgkin lymphocytic rich	Male	6 (3%)	6 (3%)	-	-	-
	Female	5 (2.5%)	5 (2.5%)	-	-	-
	Total	11 (5.5%)	11 (5.5%)	-	-	-
Classic Hodgkin nodular sclerosis	Male	75 (37.5%)	68 (34%)	7 (3.5%)	-	-
	Female	50 (25%)	47 (23.5%)	1 (0.5 %)	2 (1%)	-
	Total	125 (62.5%)	115 (57.5%)	8 (4%)	2 (1%)	
Classic Hodgkin mixed cellularity	Male	28 (14%)	28 (14%)	-	-	-
	Female	10 (5%)	10 (5%)	-	-	-
	Total	38 (19%)	38 (19%)	-	-	-
Classic Hodgkin lymphocyte depletion	Male	1 (0.5 %)	1 (0.5 %)	-	-	-
	Female	1 (0.5 %)	1 (0.5 %)	-	-	-
	Total	2 (1%)	2 (1%)	-		-
Total	Male	126 (63%)	118 (59%)	8 (4%)		-
	Female	74 (37%)	70 (35%)	1 (0.5 %)	2 (1 %)	1 (0.5 %)
	Total	200 (100%)	188 (94%)	9 (4.5%)	2 (1 %)	1 (0.5 %)

**Table2:** Distribution of gender, location, and histopathologic pattern among patients with Non-Hodgkin lymphoma.

**Pathology**	Gender		Neck	Level 1 cervical lymph nodes	Parotid gland	Buccal mucosa	Tongue	Palate	Vestibule	Gingivae	Tonsils	Maxillary sinus	Larynx	Nasopharynx	Other bones	Jaw bones
**Diffuse large** **B-cell** **lymphoma**	Male	122 (39 %)	71 (22.7%)	10 (3.2%)	4 (1.3%)	-	2 (0.6%)	4 (1.27%)	-	3 (0.9%)	8 (2.5%)	2 (0.6%)	-	7 (2. 2%)	7 (2. 2%)	4 (1.3%)
	Female	73 (23.3%)	40 (12.7%)	8 (2.5%)	3 (0.9%)	1 (0.3%)	-	2 (0.6%)	3 (0.9%)	-	2 (0.6%)	1 (0.3%)	1 (0.3%)	6 (1.9%)	4 (1.3%)	2 (0.6%)
	Total	195 (62.3%)	111 (35.4%)	18 (5.7%)	7 (2.2%)	1 (0.3%)	2 (0.6%)	6 (1.9%)	3 (0.9%)	3 (0.9%)	10 (3.2%)	3 (0.9%)	1 (0.3%)	13 (4.1%)	11 (3.5%)	6 (1.9%)
**T cell** **lymphoma**	Male	17 (5.4%)	11 (3.5%)	1 (0.3%)	1 (0.3%)	-	-	-	-	-	1 (0.3%)	1 (0.3%)	-	-	2 (0.6%)	-
	Female	9 (2.8%)	4 (1.3%)	1 (0.3%)	1 (0.3%)	-	-	-	-	-	1 (0.3%)	-	-	-	2 (0.6%)	-
	Total	26 (8.3%)	15 (4.8%)	2 (0.6%)	2 (0.6%)	-	-	-	-	-	2 (0.6%)	1 (0.3%)	-	-	4 (1.3%)	-
**Burkitt** **lymphoma**	Male	10 (3.2%)	8 (2.5%)	2 (0.63%)	-	-	-	-	-	-	-	-	-	-	-	-
	Female	9 (2.8%)	7 (2.2%)	2 (0.6%)	-	-	-	-	-	-		-	-	-	-	-
	Total	19 (6.1%)	15 (4.8%)	4 (1.3%)	-	-	-	-	-	-		-	-	-	-	-
**Follicular** **lymphoma**	Male	10 (3.2%)	5 (1.6%)	4 (1.3%)	-	-	-	-	-	-	1 (0.3%)	-	-	-	-	-
	Female	12 (3.8%)	3 (0.9%)	1 (0.3%)	2 (0.6%)	-	-	-	-	-	5 (1.6%)	-	-	1 (0.3%)	-	-
	Total	22 (7.0%)	8 (2.5%)	5 (1.6%)	2 (0.6%)	-	-	-	-	-	6 (1.9%)	-	-	1 (0.3%)	-	-
**Lymphoblastic** **lymphoma**	Male	26 (8.3%)	22 (7.0%)	2 (0.6%)	-	1 (0.3%)	-	-	-	-	-	1 (0.3%)	-	-	-	-
	Female	12 (3.8%)	12 (3.8%)	-	-	-	-	-	-	-	-	-	-	-	-	-
	Total	38 (12.1%)	34 (10.8%)	2 (0.6%)	-	1 (0.3%)	-	-	-	-	-	1 (0.3%)	-	-	-	-
**Mantle cell** **lymphoma**	Male	1 (0.3%)	-	-	1 (0.3%)	-	-	-	-	-	-	-	-	-	-	-
	Female	1 (0.3%)	1 (0.3%)	-	-	-	-	-	-	-	-	-	-	-	-	-
	Total	2 (0.6%)	1 (0.3%)	-	1 (0.3%)	-	-	-	-	-	-	-	-	-	-	-
**Mucosal** **associated** **lymphoid tissue** **lymphoma**	Male	4 (1.3%)	4 (1.3%)	-	-	-	-	-	-	-	-	-	-	-	-	-
	Female	4 (1.3%)	2 (0.6%)	-	-	1 (0.3%)	-	-	-	-	-	-	-	-	1 (0.3%)	-
	Total	8 (2.5%)	6 (1.9%)	-	-	1 (0.3%)	-	-	-	-	-	-	-	-	1 (0.3%)	-
**Anaplastic** **lymphoma**	Male	3 (0.9%)	2 (0.6%)	-	-	-	-	-	-	1 (0.3%)	-	-	-	-	-	-
	Female	-	-	-	-	-	-	-	-	-	-	-	-	-	-	-
	Total	3 (0.9%)	2 (0.6%)	-	-	-	-	-	-	1 (0.3%)	-	-	-	-	-	-
**Total**	Male	193 (61.6%)	141 (45%)	18 (5.7%)	6 (1.9%)	1 (0.3%)	2 (0.6%)	4 (1.3%)	0	4 (1.3%)	10	4 (1.3%)	0	7 (2.2%)	9 (2.8%)	4 (1.3%)
	Female	120 (38.4%)	80 (25.5%)	11 (3.5%)	6 (1.9%)	2 (0.6%)	0	2 (0.6%)	3 (0.9%)	0	8 (2.5%)	1 (0.3%)	1 (0.3%)	7 (2.2%)	7 (2.2%)	2 (0.6%)
	Total	313 (100%)	192 (61.3%)	31 (9.9%)	12 (3.8%)	3 (0.9%)	2 (0.6%)	6 (1.9%)	3 (0.9%)	4 (1.3%)	18 (5.7%)	5 (1.6%)	1 (0.3%)	14 (4.4%)	16 (5.1%)	6 (1.9%)
																

## Discussion

Numerous studies have reported epidemiological data on head and neck lymphomas (11,13). However, it seems that complementary information from different regions about various aspects of this disease could be helpful for clinicians and further investigations. According to Huh, the incidence of lymphoma exhibits marked geographic variations: lower throughout Asia and Africa and higher in North America, Australia/New Zealand, and Europe (15).

In this study, head and neck lymphomas account for 0.4% of all cancers during a 13-year retrospective study in an Iranian population. This rate has been reported as 2.5% to 11.2% in other studies (10,16). The difference might be related to the study design, data collection, socio-cultural characteristics, race, genetic, habits, and risk factors (10,15). For example, the sample size in Basirat’s study, which found head and lymphomas in 11.2% of cancer patients, was much smaller than the present report (1,510 vs. 126,450 cancer patients). 

In this study, the incidence of Hodgkin lymphoma was lower than non-Hodgkin lymphoma. This finding was in agreement with previous reports such as Basirat, Zapater, Urquhart, and Essadi (10,11,13,17). It was noted that in contrast to non-Hodgkin lymphoma the global incidence of Hodgkin lymphoma is either decreasing or stationary (18). The main reasons for decreased incidence are not clear, with only a small proportion of this decrease being attributed to the shift in the classification and the problem of misdiagnosis (19). However, Huh demonstrated that, in Korea, the incidence of Hodgkin lymphoma increased to 4.5% per year with a 7.2% increase in females and a 2.8% increase in males (15). The upward trend in women has been related to decreased parity in developed countries, implicating child bearing as a protective factor against Hodgkin lymphoma (19,20). 

Age and sex are important factors in the epidemiology of lymphomas (15). In our study, men were more commonly affected compared to women in both types of lymphomas. This finding was in accordance with Akbari, Mohtasham, and Zapater (4,5,11). Contrary to our findings, Urquhart did not find any differences between males and females (13). Furthermore, Kemp reported a higher involvement in women than in men (21). Several factors including study design, sample size, genetic and environmental discrepancies may be responsible for differences between mentioned reports (3). 

In the present study, the mean age of patients with head and neck lymphomas (46 years) was similar to previous studies (3,5,12). 

An epidemiological study of malignant lymphomas revealed that the incidence of non-Hodgkin lymphomas increased with advancing age, but in women, it decreased at the age of 80 years and older. In contrast to non-Hodgkin lymphomas, Hodgkin lymphomas exhibit specific patterns of age-incidence-curve, in association with the socioeconomic status of the studied population (15). In under developed countries, there is a peak incidence in early childhood followed by an older adult peak (over than 50 years); whereas in developed countries there is a peak incidence in young adulthood followed by an older adult peak (22,23).

According to our results (Table 1), Classic Hodgkin nodular sclerosis was the most common variation of Hodgkin disease followed by classic Hodgkin mixed cellularity and Hodgkin nodular lymphocytic predominant. These findings were in agreement with Basirat and Huh (10,15). All variations of Hodgkin lymphomas were found in the neck; whereas involvement of level 1 lymph nodes was seen in classic Hodgkin nodular sclerosis and Hodgkin nodular lymphocytic predominant. Involvement of the parotid gland only occurred in classic Hodgkin nodular sclerosis. Bone involvement was also only reported in Hodgkin nodular lymphocytic predominant. The number of affected men and women in classic Hodgkin lymphocytic depletion was equal, but in other variations there was a male predilection. On the other hand, all cases with Hodgkin lymphoma in the parotid gland and bone structures were female. 

In the present study (Table.2), diffuse large B-cell had the highest frequency among variations of non-Hodgkin lymphomas followed by lymphoblastic lymphoma, T-cell lymphoma, follicular lymphoma, and Burkitt's lymphoma. These findings were in accordance with Basirat and Zapater (10,11). Except for follicular lymphoma, in other subtypes, males were more frequently affected than females. However, there was no sex predilection in mantle cell lymphoma and MALT lymphoma. In addition, anaplastic lymphoma was not found in females. In terms of affected sites, all variations were found in the neck. Submental lymphatic (Level 1 cervical nodes) involvement was only seen in diffuse large B-cell lymphoma. Mantle cell lymphoma, mucosal associated lymphoid tissue lymphoma and anaplastic lymphoma was not found in level 1 cervical lymph nodes. Diffuse large B-cell lymphoma and T-cell lymphoma as well as follicular lymphoma were the most common variations observed in the parotid gland. In the oral cavity, tongue, palate, and vestibular mucosa were affected only by diffuse large B-cell lymphoma; whereas in the buccal mucosa diffuse large B-cell lymphoma, lymphoblastic lymphoma, and mucosal associated lymphoid tissue lymphoma were reported. All cases with tongue and vestibular mucosal involvement were males and females, respectively. There was a male predilection in the palatal lesions. Diffuse large B-cell lymphoma was the most common subtype in the tonsils followed by follicular lymphoma and T-cell lymphoma. Tonsillar diffuse large B-cell lymphoma and tonsillar follicular lymphoma were more frequent in males and females, respectively. In the maxillary sinus, diffuse large B-cell lymphoma, T-cell lymphoma, and lymphoblastic lymphoma were the most common variations and men were more frequently affected than women. Jaw bones were only involved by diffuse large B-cell lymphoma; while in other bony structures T-cell lymphoma and mucosal associated lymphoid tissue lymphoma were also found. Bone lesions were found more frequently in men than in women.

According to Mohtasham, salivary glands were more commonly affected by diffuse large B-cell lymphoma and peripheral T-cell lymphoma. Jaw bones were involved in most cases of diffuse large B-cell lymphoma, low grade B-cell lymphoma, and peripheral T-cell lymphoma. The same results were also reported for the maxillary sinus (5). In Akbari’s study, diffuse large B-cell lymphoma was the most common type of non-Hodgkin lymphoma in the tongue, palate, tonsils, and oropharynx (4). In a review article by Zucca, it was mentioned that diffuse large B-cell lymphoma was the most common variation of lymphomas in the tonsils, base of the tongue, and nasopharynx. On the other hand, it was demonstrated that the majority of salivary gland lymphomas are located in the parotid gland. Salivary gland lymphoma accounts for 5% to 10% of all salivary gland tumors (24).

As mentioned earlier, frequency and characteristics of lymphomas are affected by ethnic and environmental factors, which justify differences between our findings and previous studies. That is why the results of studies performed in an ethnical group or region cannot necessarily be generalized to other populations. In the meantime, some discrepancies in cultural, social, surrounding conditions, and geographical aspects of life as well as personal or tribal habits might influence the disease pattern. Hence, different parts of a country should be surveyed in order to obtain more comprehensive and practical statistics. Nowadays, each country is subject to complex racial variations on an extensive scale, so designing such epidemiological studies seems crucial (2,15).

## Conclusion

 Diffuse large B-cell lymphoma is the most common subtype of non-Hodgkin lymphoma especially in the tongue, palate, vestibular mucosa, and jaw bones.
